# Could high continuity of care (COC) have a negative impact on subjective health of hypertensive patients? A Japanese perspective

**DOI:** 10.1186/s12962-023-00448-6

**Published:** 2023-06-21

**Authors:** Narimasa Kumagai, Shuzo Nishimura, Mihajlo Jakovljević

**Affiliations:** 1 Faculty of Economics, Seinan Gakuin University, 6-2-92 Nishijin Sawara-Ku, Fukuoka, 814-8511 Japan; 2 Professor Emeritus of Kyoto University, Kyoto, Japan; 3 Faculty of Economics and Business Administration, Kyoto University of Advanced Science, Kyoto, Japan; 4 Institute of Advanced Manufacturing Technologies, Peter the Great St. Petersburg Polytechnic University, St. Petersburg, Russia; 5 Institute of Comparative Economic Studies, Hosei University, Chiyoda-Ku, Tokyo, Japan; 6 Department of Global Health Economics and Policy, University of Kragujevac, Kragujevac, Serbia

**Keywords:** Average treatment effects on the treated, Causal study, Hypertensive patient, Japan, Physician visit

## Abstract

**Background:**

Cardiovascular diseases, such as stroke and ischemic heart disease attributable to hypertension, are major causes of premature death in Japan and worldwide. Nevertheless, a low rate of blood pressure control among hypertensive patients has been observed in most countries. No previous studies have explored the effectiveness of physician visits among hypertensive patients in Japan.

**Methods:**

To quantify the effects of persistence in physician visits among hypertensive patients, we evaluated the causal effect of physician visits on the health of hypertensive patients. We used 16 waves of nationally representative longitudinal data drawn from the Longitudinal Survey of Middle-aged and Elderly Persons in Japan (2005–2020). To examine the causal effect of physician visits on patients’ health outcomes, we used inverse probability treatment weights and doubly robust estimation and obtained the estimates of the average treatment effects on the treated (ATETs).

**Results:**

Covariates were well balanced among patients who had physician visits during the past two consecutive years (N = 67,210; 64.9% among hypertensive patients). The estimated ATETs suggest that three consecutive years of physician visits had a negative impact on poor subjective health. Furthermore, patients without habitual exercise tended to not continue physician visits and perceived poor subjective health.

**Conclusions:**

Although the impact of frequent physician visits on blood pressure stability remains uncertain, regular appointments every 30 days can be effective for individuals with hypertension, particularly if they receive continuous instruction from their family physician. Because it is important for physicians to strengthen hypertensive patients' blood pressure control, promoting consecutive physician visits to hypertensive patients with diabetes, lower educational attainment, or smoking habits is needed.

## Introduction

Cardiovascular diseases (CVDs), such as stroke and ischemic heart disease attributable to hypertension, lead to premature death in Japan and worldwide [1–3]. Hypertension prevalence remains high in Japan; over 60% of men aged  ≥ 50 years and over 58% of women aged  ≥ 60 years had hypertension in 2016 [4]. The study by the Non-Communicable Disease (NCD) Risk Factor Collaboration used national health examination surveys from 1976 to 2017 in 12 high-income countries and observed substantial improvements in hypertension awareness, treatment, and control [5]. However, Finland, Japan, Ireland, Italy, and Spain had lower rates of awareness, treatment, and control than most other countries during the past decade.

The control rate of hypertension among hypertensive patients taking antihypertensive medication had an upward trend from 1980 to 2016 in Japan [6], but 72% (31 million) of hypertensive patients were poorly controlled in 2017. Although most developed and developing countries have observed a low rate of blood pressure control [7, 8], a recent study [9] indicated that approximately 80% of participants (N = 137) had visited a medical facility once a month during the 2017 survey. The participants in this study only had hypertension that was well-controlled with 1–2 antihypertensive drugs. Interestingly, a quarter of the participants expressed a desire to reduce their frequency of physician visits, citing the stability of their blood pressure as the main reason. This suggests that in Japan, many hypertensive patients tend to adhere to their family physicians’ treatment plans, and that regular physician visits every 30 days appear to contribute to maintaining stable blood pressure for these patients. Effective physician visits are established when the patients improve their health status using healthcare services [10], but in reality one-third of patients’ hypertension was not controlled, although patients were aware of their high blood pressure and received treatment [4]. Most patients without diabetes and taking antihypertensive medication should aim for a blood pressure level below 140/90 mmHg [11]. One reason for termination of antihypertensive therapy is the reduction in blood pressure, leading the patients to conclude that their hypertension had been cured [12]. Therefore, effective physician visits might not have been established for hypertensive patients in Japan.

As no prior studies have explored the effectiveness of physician visits among hypertensive patients in Japan, we must pay attention to patients whose hypertension are poorly controlled and evaluate the causal effect of physician visits on hypertensive patients’ health. Increased continuity of care (COC) with doctors was associated with lower mortality rates [13]. The qualitative review also reported that COC is good for people, care teams, populations, and health systems [14]. Patients’ overall health can be improved by initiating drug therapy and setting blood pressure goals [15]. However, no studies have examined whether two or three consecutive years of physician visits had a negative impact on poor subjective health. On this point, the current study contributes to the existing literature.

In general, those with a higher family income have a higher life expectancy and better self-assessed health (SAH). Measures of socioeconomic status (SES), such as income, education, and occupation, are known to correlate with mortality and morbidity [16, 17]. A positive association was found among socioeconomic positions, SAH, and the well-being of older people [18]. Many Japanese studies have also reported an association between SES and health [19–24]. Thus, we used the self-assessed health of hypertensive patients to evaluate their overall health and examined whether physician visits play a role in the patients’ health.

## Methods

### Data

We used longitudinal data over 16 consecutive years (2005–2020) obtained from the Longitudinal Survey of Middle and Elderly Persons (LSMEP) by the Japanese Ministry of Health, Labor and Welfare (MHLW). The LSMEP takes a nationally representative sample of the near elderly in Japan. The samples for this survey were randomly selected through a two-stage sampling procedure. First, 2,515 districts in 2005 were randomly selected from all 5,280 districts covered by another nationwide survey called “Comprehensive Survey of Living Conditions” conducted by the MHLW in 2004. Second, 40,877 respondents aged 50–59 years as of October 30, 2005 were randomly chosen from each selected district. A total of 34,240 individuals responded to the first survey wave (response rate: 83.8%), whereas 32,285 participants returned the questionnaires for the second wave (response rate: 92.2%). Data were collected through a combination of interviews and self-administered questionnaires. The LSMEP collects information about family situations, health status, and employment status. In the second-wave survey, the LSMEP asked about the educational attainment of both the respondents and their spouses.

### Empirical strategy

When examining the causal relationships between treatment and outcome and obtaining an unbiased assessment of the average treatment effect (ATE), researchers must pay attention to the differences in baseline characteristics between treated and untreated individuals. To account for such differences, propensity score analysis can be used. Propensity score matching is used to estimate the average treatment effects on the treated (ATETs) because the propensity score defined by [25] is the probability of an individual being assigned to a treatment condition. Researchers often use a logistic regression model and estimate the propensity score. In this model, treatment status is regressed against potential confounding variables.

Robins and Ritov proposed the doubly robust estimation that combines two approaches to estimate the causal effect of treatment on an outcome [26]. As long as either the regression model or the propensity score is specified correctly, their method that combines matching and regression is robust against misspecification of the regression function [26].

To derive the doubly robust estimator, we followed the three-step approach. First, inverse probability treatment weights (IPTW) were calculated as the inverse of the conditional probability. The IPTW creates a synthetic sample in which the distribution of the measured covariates is independent of the treatment assignment. In Eq. (1), the weight for *i*th individual (w_i_) is defined as

1 $$w_{i} \, = \,T_{i} \,/\,e_{i} \, + \,\left( {1\, - \,T_{i} } \right)\,/\,\left( {1\, - \,e_{i} } \right)$$

where T_i_ denotes whether the *i*th individual was treated (= 1) or not (= 0) and e_*i*_ denotes the propensity score. Here, using e_*i*_ as the propensity score in the weighting estimator leads to an estimator of the average treatment effect [27]. By restricting the computations of the means to a subset of treated subjects, we can obtain the ATETs. When estimating the ATETs, the weight w_*i*_ is 1 for a treated participant and (1 − T_i_)/(1 − e_*i*_) for a control participant.

Second, we used the IPTW in multivariate analysis and estimated the weighted regression models of the outcome for each treatment level. Third, the means of the treatment-specific predicted outcomes were computed.

To evaluate the propensity score distribution, the standardized difference was used to compare the mean of the continuous and binary variables between the treatment and control groups. In Eq. (2), the standardized difference for dichotomous variables is defined as

2 $${\text{Standardized}}\,{\text{difference}}\,{ = }\,\left( {Pt\, - \,Pc} \right)\,/\,\sqrt {\left\{ {Pt\,\left( {1\, - \,Pt} \right)\, + \,Pc\,\left( {1\, - \,Pc} \right)} \right\}\,/2}$$

where Pt and P*c* denote the proportions of dichotomous variables in the treated and control groups, respectively.

A standardized difference < 0.1 indicates a negligible difference in the mean or proportion of a covariate between the treatment and control groups [28]. When this condition is satisfied, we can consider that the inverse probability of weighting regression adjustment (IPWRA) estimators have the double-robust property.

## Results

The proportion of the total sum of 16 consecutive respondents to the subjects who responded to the second-wave survey in 2006 was approximately 53%. This implies that almost half of the respondents in the 2006 survey dropped out.

The samples in this study were classified as having hypertension based on a doctor’s diagnosis. Hypertension was defined as a systolic blood pressure (BP) ≥ 140 mmHg, a diastolic BP ≥ 90 mmHg, or use of antihypertensive medication. The control rate of hypertension was defined as the proportion of those with a systolic BP < 140 mmHg and a diastolic BP < 90 mmHg among hypertensive patients taking antihypertensive medication [6].

Table 1 shows the differences in key variables of hypertensive patients, such as SAH between the two groups. Sample characteristics of the elderly not having hypertension are shown in Table 5. The original SAH was recorded and evaluated on a scale of 1–6 (1 = poor, 6 = excellent). This study used the sample that excluded non-respondents of SAH. Among individuals who responded to gender, marital status, and educational attainment, older individuals with lower educational attainment tended not to respond to their SAH (see Table 6). In contrast, the elderly with higher educational attainment tended to respond to their SAH.

**Table 1:** Sample characteristics of hypertensive patients classified by physician visits. Sources: Longitudinal Survey of Middle-aged and Elderly Persons 2005–2020

Variables	Patients who continued physician visits during the past two consecutive years			Patients who did not continue physician visits during the past two consecutive years		
	*N*	Mean	SD	*N*	Mean	SD
Self-assessed health	72,613	3.948	0.90	43,915	3.882	0.94
Dummy variable for poor self-assessed health	72,613	0.058	0.23	43,915	0.072	0.26
Dummy variable for good self-assessed health	72,613	0.253	0.43	43,915	0.244	0.43
Demographic variables						
Age	72,613	64.25	4.60	43,915	61.36	5.39
Gender (male = 1)	72,613	0.525	0.50	43,915	0.551	0.50
Married (reference)	72,613	0.939	0.24	43,915	0.922	0.27
Never married	72,613	0.061	0.24	43,915	0.067	0.25
Divorced or widowed	72,613	0.000	0.01	43,915	0.011	0.10
Dummy variable for living together with family members excluding spouse	72,578	0.542	0.50	43,794	0.585	0.49
Dummy variable for earned income during the past month	62,583	0.596	0.49	36,551	0.715	0.45
Objective health status						
Dummy variable for having diabetes	71,512	0.184	0.39	43,607	0.161	0.37
Dummy variable for having lipidemia	71,131	0.272	0.45	43,347	0.212	0.41
Dummy variable for having stroke	70,612	0.043	0.20	43,221	0.047	0.21
Dummy variable for having heart diseases	71,245	0.090	0.29	43,492	0.085	0.28
Dummy variable for having cancer	70,919	0.038	0.19	43,310	0.032	0.18
Lifestyle						
Almost every day drinking	72,613	0.236	0.42	43,915	0.232	0.42
No habitual exercise	72,396	0.338	0.47	43,352	0.389	0.49
Smoking habit	72,613	0.161	0.37	43,915	0.219	0.41
Educational attainment						
Junior high school	72,613	0.173	0.38	43,915	0.202	0.40
High school (reference)	72,613	0.511	0.50	43,915	0.493	0.50
Vocational school or junior college	72,613	0.142	0.35	43,915	0.139	0.35
University or graduate school	72,613	0.168	0.37	43,915	0.159	0.37

Non-respondents of self-assessed health are excluded

The mean SAH among the hypertensive patients who continued physician visits during the past two consecutive years (HPCPV) was approximately 3.948, which was larger than that of the comparison group (3.882). In contrast, the prevalence of poor SAH (PSAH; SAH = 1 or 2) in HPCPV was lower than that in hypertensive patients without continuing physician visits (0.058 < 0.072). The proportion of having cancer, diabetes, heart diseases, lipidemia, and stroke among elderly with hypertension was larger than that in the comparison group (see Table 5). The means between the two groups were significantly different at the 1% level.

The mean SAH among the elderly who did not have diabetes or lipidemia was > 4 (4.003, N = 71,081). However, the mean SAH among the elderly with both diabetes and lipidemia was < 3.5 (3.489, N = 6,934). Thus, we must pay attention to diabetes and lipidemia when splitting hypertensive patients.

Table 2 shows the relationship between SAH and the patients’ feeling regarding symptoms of high blood pressure. If hypertensive patients felt that their symptoms (FS) were worse than their onsets during the past year, the FS took a value of 1. More than half of the respondents felt unchanged compared with their onsets (FS = 2). Almost 20% of this group responded that their SAH was good or excellent (SAH = 5 or 6, respectively). By contrast, the prevalence of PSAH in this group was 7%. We can infer that most hypertensive patients tended not to respond that their SAH was good or excellent when they felt their symptoms were better than their onsets during the past year (FS = 3).

**Table 2:** Relationships between patients’ self-assessed health and feelings of symptoms. Sources: Longitudinal survey of middle-aged and elderly persons; 2005–2020

FS	Self-assessed health						
	1	2	3	4	5	6	Total
1	150	232	431	213	44	5	1,075
	0.14	0.22	0.41	0.2	0.04	0	1.02
2	714	3,534	14,617	26,673	10,871	871	57,280
	0.68	3.35	13.85	25.28	10.3	0.83	54.28
3	257	1,450	7,185	23,669	13,115	1,490	47,166
	0.24	1.37	6.81	22.43	12.43	1.41	44.7
Total	1,121	5,216	22,233	50,555	24,030	2,366	105,521
	1.06	4.94	21.07	47.91	22.77	2.24	100

FS = 1 (= 3); Felt worse (better) symptoms of high blood pressure than its onset during the past year

To identify the determinants or potential confounders of physician visits among hypertensive patients, assuming the dependent variable distributed as Bernoulli, we estimated three generalized linear models with a logit link function by classification. Table 3 lists the estimation results. Notably, diabetes and lipidemia had negative effects on physician visits among patients who had physician visits during the past two consecutive years. In contrast, male patients and those having diabetes, with lower educational attainment, without habitual exercise, or with smoking habits tended not to have physician visits during the past two consecutive years. We can conjecture that these factors are associated with a low rate of blood pressure control among hypertensive patients taking antihypertensive medication.

**Table 3:** Generalized linear models with logit link function

Dependent variable	Physician visits among hypertensive patients		
	The past two consecutive years		All
Variables	Yes	No	
Diabetes	−0.0934 (0.122)	0.541*** (0.0422)	0.503*** (0.0419)
Lipidemia	0.0524 (0.111)	−0.426*** (0.0317)	−0.408*** (0.0325)
Felt worse symptoms of high blood pressure than its onset during the past year	−0.0230 (0.505)	0.253 (0.192)	−0.248 (0.198)
Physician visits during the past year			2.786*** (0.0541)
Physician visits during the past two consecutive years			0.916*** (0.104)
Physician visits during the past three consecutive years	0.539*** (0.111)		0.490*** (0.107)
Age	0.0219** (0.0112)	0.0203*** (0.00282)	0.0465*** (0.00298)
Gender (male = 1)	−0.201* (0.107)	−0.435*** (0.0315)	−0.385*** (0.0320)
Never married	−0.217 (0.182)	−0.0915* (0.0523)	0.112** (0.0533)
Divorced or widowed	−2.881*** (1.060)		0.688 (1.121)
Living together with family members excluding spouse	0.0796 (0.0974)	0.0636** (0.0285)	0.0524* (0.0289)
Drinking habit (lagged)	−0.153 (0.112)	0.00519 (0.0339)	0.00450 (0.0343)
No habitual exercise (lagged)	-−0.115 (0.101)	0.111*** (0.0293)	0.103*** (0.0299)
Smoking habit (lagged)	−0.161 (0.125)	−0.228*** (0.0340)	−0.216*** (0.0351)
Junior high school	−0.0895 (0.132)	0.342*** (0.0401)	0.314*** (0.0405)
Vocational school or junior college	−0.218 (0.139)	0.0606 (0.0428)	0.0596 (0.0434)
University or graduate school	0.0371 (0.140)	−0.0295 (0.0380)	−0.0172 (0.0391)
Constant term	3.427*** (0.718)	0.441** (0.183)	−1.840*** (0.194)
*N*	67,210	36,145	103,361

Standard errors in parentheses. *** p < 0.01, ** p < 0.05, * p < 0.1

We used patients who had physician visits during the past two consecutive years (N = 67,210; 64.9% among hypertensive patients) and estimated the SAH function. Because variables that were present after treatment assignment should not be included in the propensity score model, we used lagged lifestyle variables. This specification satisfied the balance test after matching. Table 6 shows that the proportion of the treated group after matching is 0.501, and all standardized ratios shown in the weighted series are below 0.1. The covariates were well balanced through inverse probability weighting, although the proportion of the raw treated group was 0.993 (65,626/66,064). In contrast, the imbalanced covariates among all hypertensive patients are shown in Table 7, which indicates that covariates among hypertensive patients who did not continue physician visits during the past two years were imbalanced when using the same specification.

Table 4 illustrates the doubly robust estimation of physician visits. The estimated ATETs suggest that current physician visits had a negative impact on the PSAH at the 1% significance level. Older ages, being divorced or widowed, and having higher educational attainment tended to have negative effects on the PSAH. In contrast, patients without habitual exercise tended to not continue physician visits and perceived poor subjective health. Therefore, it is considered that three consecutive years physician visits contribute to the prevention of health deterioration among hypertensive patients. Habitual exercise is important for patients who prefer consecutive physician visits. However, the current physician visits have not shown positive impacts on SAH.

**Table 4:** The doubly robust estimation of physician visits

Covariates of the doubly robust estimation			
Dependent variables	Physician visits among hypertensive patients	Poor self-assessed health (PSAH)	
Physician visits among hypertensive patients (ATET)			−0.0358*** (0.0128)
Physician visits during the past three consecutive years	0.550*** (0.112)		
Age	0.0188* (0.0108)	−0.00266 (0.00335)	−0.000533*** (0.000196)
Gender (male = 1)	−0.146 (0.113)	0.00859 (0.0320)	0.00309 (0.00206)
Never married	−0.226 (0.178)	−0.0808** (0.0316)	0.00142 (0.00357)
Divorced or widowed		0.871*** (0.0673)	−0.0963*** (0.0250)
Living together with family members excluding spouse	0.0806 (0.0991)	−0.0186 (0.0276)	−0.00299* (0.00171)
Drinking habit (lagged)	−0.00007 (0.117)	−0.0170 (0.0310)	0.00205 (0.00207)
No habitual exercise (lagged)	−0.200** (0.101)	0.0236 (0.0291)	0.0240*** (0.00195)
Smoking habit (lagged)	−0.192 (0.124)	0.0133 (0.0340)	0.00164 (0.00247)
Junior high school	−0.0488 (0.135)	0.0104 (0.0416)	0.0204*** (0.00268)
Vocational school or junior college	−0.245* (0.141)	−0.0337 (0.0343)	−0.00105 (0.00247)
University or graduate school	−0.000704 (0.141)	0.0121 (0.0635)	−0.00610 (0.00470)
Gender × University or graduate school		−0.131* (0.0747)	−0.0140*** (0.00522)
Diabetes		0.0184 (0.0378)	0.0494*** (0.00289)
Stroke		0.200* (0.113)	0.105*** (0.00749)
Heart diseases		0.146* (0.0792)	0.0859*** (0.00539)
Cancer		0.235** (0.114)	0.136*** (0.00819)
Diabetes × Heart diseases		0.298* (0.153)	0.0126 (0.0110)
Felt worse symptoms of high blood pressure than its onset during the past year		0.0679(0.131)	0.228*** (0.0188)
Constant term	3.594*** (0.704)	0.242 (0.220)	0.0493*** (0.0130)
*N*	66,064	66,064	
Sample		PSAH = 0	PSAH = 1

Robust standard errors in parentheses

*** p < 0.01, ** p < 0.05, * p < 0.1

## Discussion

Noncommunicable chronic diseases (NCDs) with hypertension and diabetes as prominent example, continue to shape entire morbidity landscape in industrialized aging nations ranging from Japan to Europe [29, 30]. Increased primary care physician visits among patients with hypertension were associated with a lower risk of death, but an increased risk of hospitalization [31]. Although previous studies have not revealed effective physician visits, the current study attempted to examine the causal effect of physician visits on health outcomes in hypertensive patients. To obtain estimates of the ATETs, we used inverse probability treatment weights and doubly robust estimation. Contrary to a recent study using propensity score matching [32], we found that educational history influenced patients' behavior regarding the preference for having physician visits.

The estimated ATETs suggest that three consecutive years of physician visits had a negative impact on poor subjective health. The results also showed that (1) patients without habitual exercise tended not to continue physician visits and perceived poor subjective health; (2) having diabetes or lipidemia had negative effects on physician visits among the patients who had physician visits during the past two consecutive years. The former result coincides with evidence that individuals with regular physical activities (RPA) exhibit greater persistence of latent health stock (LHS) than individuals without RPA [33]. Because two causal relationships were considered between smoking habits, RPA, and LHS—a flow to RPA from smoking habits and a flow to LHS from RPA [33]–smoking cessation is important to increase habitual exercise. The latter result may indicate that hypertension without habitual exercise was not associated with greater persistence of physician visits.

Although the impact of frequent physician visits on blood pressure stability remains unclear, it appears that physician visits that occur less frequently than twice a month or with intervals longer than 40 days may not be effective for hypertensive patients. To maintain stable blood pressure in hypertensive patients, receiving continual instruction from their family physician is important. The relatively larger impact of the patients' heterogeneity may be attributed to their physician visits [34]. To ensure effective physician visits, primary care physicians should encourage hypertensive patients to engage in regular exercise and schedule consecutive follow-up appointments. This study suggests that to achieve effective physician visits, primary care physicians in Japan should recommend habitual exercise and consecutive visits to hypertensive patients. As shown in [32], receiving medical treatment is associated with lower systolic blood pressure. Although encouraging physician visits after screening might be a weak intervention [32], it is important for physicians to strengthen hypertensive patients' blood pressure control because consecutive physician visits are associated with patients' blood pressure control. Thus, physicians should promote consecutive physician visits to hypertensive patients with diabetes, lower educational attainment, or smoking habits [35].

In Japan, the duration of doctor consultations over the past year was found to be positively associated with PSAH among middle-aged individuals, regardless of gender [36]. However, middle-aged women who had multiple roles as mothers or wives tended to rate their health lower compared to men. Additionally, middle-aged or elderly women who dedicated significant time to domestic work were less likely to benefit from marriage compared to men who had lifestyle diseases [37]. On the other hand, the empirical results of this study indicated that being divorced or widowed had a strong negative impact on PSAH, while age had a relatively minor negative effect (Table 4). This suggests that some retired individuals who continued to visit physicians were less likely to evaluate their subjective health as poor compared to other elderly or middle-aged individuals. As widowed individuals, particularly women, comprised a significant portion of this group, older widowed women may not tend to perceive their health as poor. Therefore, a decrease in domestic responsibilities and an increase in time devoted to physician visits appear to have a positive influence on the subjective health of older widowed women. The deterioration of hypertensive patients' physical condition increases the number of patients with cerebrovascular and heart diseases, leading to increased costs of long-term care and health care expenses. The downward trend in the labor force in developed countries implies that securing the future financial resources of healthcare and long-term care becomes difficult. It is unlikely that we shall be able to tackle NCD’s burden effectively in near future [38, 39]. Therefore, from the viewpoint of cost containment of future healthcare and long-term care, physicians must pay much attention to hypertensive patients with risky health behaviors.

When comparing two groups of hypertensive patients, it is important to consider that selection bias could be associated with imbalanced covariates among those who did not continue physician visits in the past two years. Lower continuity of care might be a result of complications from lifestyle diseases, such as neurological symptoms in hypertensive patients with diabetes or other chronic conditions. As a result, there could be significant heterogeneity in the selection of physician visits among hypertensive patients. Researchers should account for the magnitude of selection bias when measuring the true positive treatment effect [40]. In future studies, it is crucial to examine the development of complications in hypertensive patients with diabetes or other chronic diseases and investigate the causal effect of physician visits on the health of hypertensive patients who have not continued regular visits in the past two years.

## Conclusions

High COC with doctors had a negative impact on poor subjective health of hypertensive patients. Although the impact of frequent physician visits on blood pressure stability remains uncertain, regular appointments every 30 days have shown effectiveness for hypertensive patients when accompanied by continual instruction from their family physician. In order to enhance blood pressure control in hypertensive patients, it is crucial for physicians to promote consecutive physician visits, particularly for those with comorbidities such as diabetes, lower educational attainment, or smoking habits.

## Data Availability

The datasets used were provided by the Japanese Ministry of Health, Labor and Welfare. Our data cannot be shared with any third party.

## Appendix

See Tables 5, 6, 7

**Table 5:** Sample characteristics of the elderly not having hypertension. Sources: Longitudinal Survey of Middle-aged and Elderly Persons 2005–2020

Elderly not having hypertension			
Variables	*N*	Mean	SD
Self-assessed health	242,699	4.237	0.92
Dummy variable for poor self-assessed health	242,699	0.039	0.19
Dummy variable for good self-assessed health	242,699	0.390	0.49
Demographic variables			
Age	242,699	61.08	5.28
Gender (male = 1)	242,699	0.452	0.50
Married (reference)	242,699	0.912	0.28
Never married	242,699	0.079	0.27
Divorced or widowed	242,699	0.009	0.09
Dummy variable for living together with family members excluding spouse	242,243	0.591	0.49
Dummy variable for earned income during the past month	200,983	0.703	0.46
Objective health status			
Dummy variable for having diabetes	241,975	0.089	0.28
Dummy variable for having lipidemia	241,654	0.137	0.34
Dummy variable for having stroke	241,345	0.015	0.12
Dummy variable for having heart diseases	241,771	0.038	0.19
Dummy variable for having cancer	241,513	0.034	0.18
Lifestyle			
Almost every day drinking	242,699	0.282	0.45
No habitual exercise	240,491	0.366	0.48
Smoking habit	242,699	0.213	0.41
Educational attainment			
Junior high school	242,699	0.156	0.36
High school (reference)	242,699	0.494	0.50
Vocational school or junior college	242,699	0.173	0.38
University or graduate school	242,699	0.170	0.38

Non-respondents of self-assessed health are excluded

**Table 6:** Determinants of non-response of SAH

Variables	Response/Non-response
Age	−0.0190*** (0.00117)
Gender (male = 1)	0.0248** (0.0124)
Never married	−0.0375 (0.0232)
Divorced or widowed	0.0297 (0.0850)
Living together with family members excluding spouse	0.00941 (0.0121)
Junior high school	−0.166*** (0.0149)
Vocational school or junior college	0.0116 (0.0176)
University or graduate school	0.0608*** (0.0189)
Constant term	3.507*** (0.0760)
*N*	394,812

Standard errors in parentheses

*** p < 0.01, ** p < 0.05, * p < 0

**Table 7:** Covariate balance tests after matching (Doubly robust estimation)

	Raw	Weighted
*N*	66,064	66,064
Treated	65,626	33,068
Control	438	32,996

	Standardized difference		Variance ratio	
	Raw	Weighted	Raw	Weighted
Age	0.18	0.00	0.98	1.04
Gender (male = 1)	−0.08	−0.01	1.01	1.00
Married	0.09	0.01	0.75	0.98
Never married	−0.08	0.00	0.76	1.01
Living together with family members excluding spouse	0.01	−0.01	1.00	1.00
Diabetes	−0.02	−0.03	0.96	0.95
Lipidemia	0.04	0.04	1.05	1.05
Stroke	0.01	0.00	1.07	0.99
Heart disease	−0.02	−0.05	0.94	0.87
Cancer	−0.02	−0.05	0.90	0.81
Drinking habit (lagged)	−0.03	−0.06	0.96	0.93
No habitual exercise (lagged)	−0.10	−0.02	0.94	0.98
Smoking habit (lagged)	−0.11	−0.01	0.83	0.99
Junior high school	−0.01	0.01	0.97	1.03
High school	0.06	0.01	1.00	1.00
Vocational school or junior college	−0.06	−0.01	0.89	0.99
University or graduate school	0.00	0.00	1.01	0.99

## Abbreviations

ATE: Average treatment effect
ATETs: Average treatment effects on the treated
BP: Blood pressure
COC: Continuity of care
CVDs: Cardiovascular diseases
FS: Felt that hypertensive patients’ symptoms
HPCPV: Hypertensive patients who continued physician visits during the past two consecutive years
IPTW: Inverse probability treatment weights
IPWRA: Inverse probability of weighting regression adjustment
LHS: Latent health stock
LSMEP: Longitudinal survey of middle and elderly persons
MHLW: Ministry of health, labor and welfare
NCD: Non-communicable disease
PSAH: Poor self-assessed health
RPA: Regular physical activities
SAH: Self-assessed health
SES: Socioeconomic status
